# MicroRNA-223-3p regulates allergic inﬂammation by targeting INPP4A

**DOI:** 10.1016/j.bjorl.2020.05.020

**Published:** 2020-06-25

**Authors:** Yong Zhou, Ting Zhang, Yongbing Yan, Bo You, Yiwen You, Wei Zhang, Jing Chen

**Affiliations:** a Affiliated Hospital of Nantong University, Institute of Otolaryngology Head and Neck Surgery, Nantong, China; b Affiliated Hospital of Nantong University, Department of Otolaryngology Head and Neck Surgery, Nantong, China

**Keywords:** Allergic rhinitis, Allergic inflammation, MicroRNA-223-3p, INPP4A

## Abstract

**Introduction:**

Emerging evidence indicates that physiological and pathological conditions of the nose are posttranscriptionally regulated by microRNAs, a class of small noncoding RNAs. Recently, microRNA-223-3p has been increasingly implicated in the modulation of allergic rhinitis

**Objective:**

This study aimed to assess the role and mechanism of microRNA-223-3p in a mouse model of allergic rhinitis.

**Methods:**

The expression level of miR-223-3p was measured in the serum of 41 allergic rhinitis patients and 39 healthy controls using quantitative real time polymerase chain reaction. BALB/c mice were used to establish an allergic rhinitis model by intraperitoneal sensitization and intranasal challenge with ovalbumin. MicroRNA-223-3p agomir/antagomir was then intranasally administered to mice after ovalbumin challenge for another week. The symptoms of nasal rubbing and sneezing were recorded. Serum ovalbumin-specific immunoglobulin E concentration, microRNA-223-3p expression and proinflammatory cytokine (IL-4, IL-5, IFN-γ) levels in nasal mucosa were measured by ELISA and quantitative real time polymerase chain reaction, respectively. Histopathologic changes were evaluated using hematoxylin and eosin staining.

**Results:**

MicroRNA-223-3p levels increased significantly in both allergic rhinitis patients and allergic rhinitis mice. In addition, upregulation of microRNA-223-3p levels by nasal administration of microRNA-223-3p agomir also markedly increased the concentration of ovalbumin -specific IgE, the frequencies of nasal rubbing and sneezing, the levels of proinflammatory cytokines (IL-4, IL-5, IFN-γ) and eosinophil infiltration in the nasal mucosa of allergic rhinitis mice. Moreover, microRNA-223-3p antagomir appeared to strongly ameliorate the symptoms and pathology in nasal mucosa. Subsequently, we demonstrated for the first time that microRNA-223-3p negatively regulated INPP4A expression by binding with the 3′ untranslated region (3′UTR) of INPP4A.

**Conclusions:**

These findings indicate that microRNA-223-3p plays an important role in regulating the pathology and symptoms of allergic rhinitis by targeting INPP4A.

## Introduction

In some individuals with a genetic predisposition, factors such as allergens, infections and pollution may trigger abnormal immune responses, resulting in allergic airway diseases such as Allergic Rhinitis (AR).^1^ AR is thought to be an allergen-specific IgE-mediated immune response and is characterized by increased T-helper 2 (Th2) cytokine production, excessive eosinophil infiltration and mucus secretion.^2^, ^3^ Among the most common diseases globally, AR causes noninfectious inflammation of the nasal mucosa and hypersensitivity. Brozek et al. reported that the prevalence of self-reported AR was approximately 2%–25% in children, and the confirmed rate of AR in adults in Europe ranged from 17% to 28.5%.^4^ The main clinical manifestations of AR include rhinorrhea, itching, sneezing and nasal congestion, which may cause sleep disturbances and decreased attention, seriously impacting patients’ quality of life as well as school and work performance.^5^, ^6^ Furthermore, AR is the basis of many complications, such as sinusitis, nasal polyps, allergic conjunctivitis, pharyngitis, otitis media, tracheitis, bronchitis and asthma.^7^ Asthma has been estimated to occur in 15%–38% of patients with AR, and AR is a risk factor for asthma. In addition, uncontrolled moderate-severe AR impacts asthma control.^4^ Medical costs caused by asthma are substantial, and indirect costs associated with poor productivity are even more extensive. Published data have revealed that the incidence of AR is growing at a rapid rate in recent decades.^4^

The mechanism underlying the occurrence of AR is complicated. During the past several decades, it has generally been considered that AR is the pathologic consequence of the activation and infiltration of mast cells, eosinophils, and basophils, inappropriate or exaggerated type 2 immune responses, and the generation and release of various inﬂammatory mediators.^8^ The mainstream clinical therapy for AR consists of disease control with antihistamines, leukotriene receptor antagonists, mast cell stabilizers, allergen-specific immunotherapy, intranasal corticosteroids and decongestants.^9^ Unfortunately, the molecular mechanisms mediating AR remain obscure. Although the above drugs relieve symptoms in most patients, they do not cure the disease. Moreover, 20% of AR patients receiving therapy already show insufficient subjective and objective improvement.^10^ It is worth mentioning that immunotherapy is still limited to mite-specific AR patients. Even if it works, the possibility of recurrence still exists. In addition, not all participants finish the entire course of treatment. Therefore, it is urgently necessary to further understand the pathogenesis of AR and develop safe new therapeutics that can resolve the disease.

MicroRNAs (miRNAs), known as critical regulatory molecules, are a large class of highly conserved short, single-stranded, noncoding RNA molecules of 19–25 nucleotides in length encoded by endogenous genes.^11^ They can induce posttranscriptional gene silencing by binding to the 3′Untranslated Region (3′UTR) of their target messenger RNAs (mRNAs), resulting in mRNA degradation or translational repression.^12^ Furthermore, a single miRNA can have thousands of potential targets by binding to their target mRNAs through complementary sequences of 6–8 nucleotides in length.^11^, ^13^ Moreover, due to their high stability and relatively long lifetime, miRNAs are very attractive disease biomarkers.^14^, ^15^ Produced by a variety of cells, miRNAs can be secreted into body fluids and play a key role in a wide variety of main functions in most cells, such as the cell cycle, proliferation, differentiation, apoptosis, and the immune response, through the regulation of multiple target genes.^12^, ^16^, ^17^ Recent studies have shown that miRNAs are critical in the pathogenesis of immune and inflammatory diseases, including AR.^18^, ^19^ Specifically, increasing evidence suggests that microRNA-223-3p (miR-223-3p) may be involved in allergic inflammation, which is an important manifestation of the pathogenesis of AR.^18^, ^20^ Hence, in this study, we explored whether and how miR-223-3p regulates allergic inflammation by establishing a mouse model of AR. We aimed to determine the miR-223-3p expression level in AR and the underlying molecular mechanisms of this disease in vivo, thereby proposing a potential therapeutic alternative.

## Methods

### Human AR specimens and ethics statement

Forty-one patients with AR and thirty-nine healthy control subjects were enrolled in the current study. AR was diagnosed according to the Allergic Rhinitis and its Impact on Asthma (ARIA) Guidelines (2016 Revision) after confirmation of disease history, nasal symptoms, clinical examination and skin prick test.^4^ The healthy control subjects did not have nasal diseases or a history of asthma. Details of the subjects’ characteristics are included in Table 1. Peripheral blood samples collected from the participants were immediately centrifuged to isolate the serum, from which total RNA was extracted for real-time PCR. The study was approved by the ethics committee of our institution, and written informed consent was obtained from each participant (Research Number: 2019-L062).

**Table 1:** Clinical features of healthy controls and AR patients.

Characteristic	AR	Healthy control
Case (nº)	41	39
Gender (male/female)	25/16	21/18
Age (year)	15.9 ± 21.2	16.5 ± 20.5
Duration (year)	2.7 ± 1.2	‒
History of asthma (%)	4.9%	‒
Family history (Yes/No)	2/39	‒
Food allergy history (Yes/No)	5/36	‒
INSS score		
Nasal rhinorrhea	2.5 ± 0.7	‒
Itching	2.3 ± 0.4	‒
Sneezing	2.2 ± 0.6	‒
Congestion	2.1 ± 0.7	‒
TNSS score	8.5 ± 1.2	‒

INSS, Individual Nasal Symptom Score; TNSS, Total Nasal Symptom Score.

### Animals

Thirty 8 week-old BALB/c mice were obtained from the Experimental Animal Center of our university and housed in a specific-pathogen-free biohazard containment facility maintained at a 12 h dark/light cycle and 20° ± 1 °C room temperature with free access to food and water. All animal experiments conducted in this study followed the NIH Guidelines and were approved by the Administration Committee of Experimental Animals of our institution (Research Number: 20180529-001).

### AR model production

**Figure 1:**
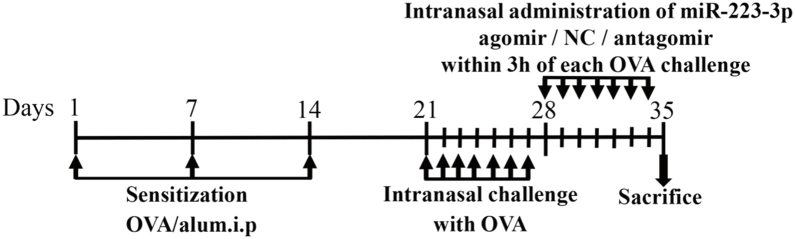
Schematic diagram of the animal experiment. BALB/c mice were intraperitoneally injected with Ovalbumin (OVA) and aluminum hydroxide (alum) on days 1, 7, and 14 to promote primary sensitization. One week after the final intraperitoneal injection, mice were intranasally challenged with OVA for a week to induce secondary immunization, followed by intranasal treatment using mismatched agomir/miR-223-3p agomir/mismatched antagomir/miR-223-3p antagomir daily within the 3 h of each OVA challenge for 7 days.

The mice were divided into six groups: control, AR (AR mice treated with PBS), mismatched agomir (AR mice treated with mismatched agomir), miR-223-3p agomir (AR mice treated with miR-223-3p agomir), mismatched antagomir (AR mice treated with mismatched antagomir) and miR-223-3p antagomir (AR mice treated with miR-223-3p antagomir). The AR model was induced according to the protocol summarized in Fig. 1.^21^ In short, mice were sensitized with intraperitoneal injection of 200 μL of saline containing 25 μg ovalbumin (OVA; grade V; Sigma-Aldrich, St. Louis, MO, USA) and 2 mg of aluminum hydroxide gel on days 1, 7 and 14. One week after the primary sensitization, 3% OVA diluted in 20 μL of saline was dripped into the nasal cavities of mice daily from day 21 to day 27 for secondary immunization. For the control (normal) group, only saline was given for intraperitoneal injection and intranasal challenge from beginning to end.

### Treatment with an intranasal administration of MiR-223-3p agomir or antagomir

MiR-223-3p agomir, miR-223-3p antagomir, mismatched agomir and mismatched antagomir were synthesized at RiboBio Company (Guangzhou, China). As shown in Fig. 1, mice were treated with an intranasal administration of 5 pmoL/μL miR-223-3p agomir or miR-223-3p antagomir diluted in 20 μL saline within 3 h of OVA challenge daily on days 28‒34. The mismatched agomir/mismatched antagomir groups of mice were intranasally administered 5 pmoL/μL mismatched agomir/mismatched antagomir diluted in 20 μL saline, while the control group was treated with only saline.

### Symptom score

Five minutes after the final OVA challenge on day 27, the frequencies of sneezing and nasal rubbing were recorded by a blinded observer twice at 15 min intervals.^21^, ^22^ Scores were calculated as follows: 1 ‒ Slightly rubs the nose or sneezes less than 3 times; 2 ‒ Rubs the nose repeatedly or sneezes between 3 and 10 times; and 3 ‒ Rubs nose and face seriously or sneezes more than 11 times. Mice with scores greater than 4 were further treated.

### Tissue preparation

Mice were euthanized 24 h after the final intranasal challenge. For RT-PCR, the nasal mucosa of the mice was obtained, immediately immersed in liquid nitrogen and then stored at −80 °C until use. For histological analyses, the heads of the mice were removed and then fixed in 4% paraformaldehyde for 24 h at 4 °C. Nasal tissues of the mice were decalcified, dehydrated in increasing concentrations of ethanol, and then embedded in paraffin.

### Quantitative real-time RT-PCR (qRT-PCR) analysis

Total RNA was extracted from cells or the nasal mucosa tissues of the mice with TRIzol reagent (Invitrogen, Carlsbad, CA, USA), and then the concentration and purity of RNA were determined. Serum miRNA was isolated using the miRcute Serum/plasma miRNA isolation kit (TIANGEN Biotech, Beijing, China) according the manufacturer’s instructions. Two micrograms of RNA was reverse transcribed to cDNA, and qRT-PCR was performed according to the instructions for the Power SYBR Green PCR Master Mix (Applied Biosystems, USA). The specific target gene primers are listed in Table 2. The reaction conditions for qRT-PCR were as follows: predenaturation at 95 °C for 10 min, followed by 40 amplification cycles at 95 °C for 20 s, annealing at 62 °C for 30 s and extension at 72 °C for 30 s. For qualification of the expression level of miR-223-3p, the synthetic spike control cel-miR-39 was used as an invariant control for the serum miRNA, while U6 served as the corresponding internal reference for cellular miRNA. The average transcript levels of IL-4, IL-5 and IFN-γ in nasal mucosa and cellular INPP4A were normalized to GAPDH levels. The data were calculated using the 2^−ΔΔCT^ method.

**Table 2:** The primer sequences used for quantitative PCR.

Genes		Primers sequences (5′ to 3′)
miR-223-3p	‒	UGUCAGUUUGUCAAAUACCCCA
IL-4	Forward	CAAACGTCCTCACAGCAACG
	Reverse	AGGCATCGAAAAGCCCGAAA
IL-5	Forward	AAATTCCTGTAGCGCAGGCT
	Reverse	ACCCTGATGCAACGAAGAGG
IFN-γ	Forward	AGCCTCAGGAAGCGGAAAAG
	Reverse	CTCATTGAATGCTTGGCGCT
INPP4A	Forward	AAAGTGTGGCTGAACGTGGA
	Reverse	GCTGTCCGGGTTACCTTTCT
GAPDH	Forward	GGTTGTCTCCTGCGACTTCA
	Reverse	CCCTAGGCCCCTCCTGTTAT
U6	Forward	CGCTTCGGCAGCACATATACTAAAATTGGAAC
	Reverse	GCTTCACGAATTTGCGTGTCATCCTTGC

### Cell culture and MiRNA transfection

We purchased HEK293T cells from the Cell Bank of Chinese Academy of Medical Science (Shanghai, China) and maintained them in RPMI-1640 medium containing 10% FBS, 100 U/mL penicillin and 100 U/mL streptomycin (Invitrogen) in a humidified incubator with 5% CO_2_ at 37 °C. In the different groups, transfection was performed using miR-223-3p mimics or miR-223-3p inhibitor or their corresponding negative controls (GeneChem, Shanghai, China) diluted with Opti-MEM I medium (Invitrogen) and transfected into HEK293T cells with Lipofectamine 2000 (Invitrogen) according to the manufacturer’s instructions.

### Quantitative measurement of OVA-specific immunoglobulin E (IgE)

To evaluate allergic reactions, serum levels of OVA-specific IgE were measured using solid-phase ELISA kits (CUSABIO, Wuhan, China). Procedures were performed according to the manufacturer’s instructions. Briefly, serum samples were harvested from mice at the time of death and then pipetted into 96 well plates precoated with purified anti-mouse IgE mAb. Anti-mouse IgE-conjugated Horseradish Peroxidase (HRP) was added to the plates. The reactions were developed using 3, 3′, 5, 5′-tetramethylbenzidine and terminated by adding H2SO4. The Optical Density (OD) was recorded by a luminometer set at 450 nm.

### Histological analyses

For the evaluation of nasal histology, approximately 4 µm thick sections coronally sectioned from the nasal vestibule were stained with Hematoxylin and Eosin (H&E). The number of eosinophils in the nasal mucosa was counted under a light microscope (×400 magnification).

### Statistical analysis

All data were analyzed with GraphPad Prism software (version 8.3; GraphPad Software, Inc., La Jolla, CA, USA) and are expressed as the mean ± Standard Deviation (SD). Correlations among the four groups were calculated using one-way analysis of variance followed by Tukey’s multiple comparison post hoc tests, *p* < 0.05 was considered statistically significant.

## Results

### Increased expression of miR-223-3p in the serum of patients with AR

**Figure 2:**
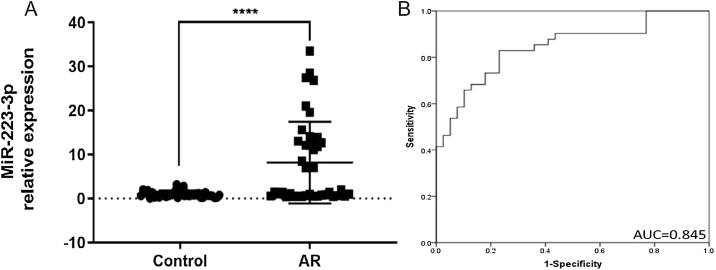
MiR-223-3p is highly expressed and serves as a clinical biomarker in AR patients. (A) The expression level of miR-223-3p in AR patients (n = 41) was significantly higher than that in healthy participants (n = 39). (B) ROC curve analysis of the diagnosis of AR for the miR-223-3p marker. AUC: area under the curve. Statistical significance was assessed using two-tailed Student’s *t*-test. The error bar indicates the SD. (****p < 0.0001).

The main clinical features of healthy controls and AR patients are shown in Table 1. In our results, the expression level of miR-223-3p in AR serum was significantly higher than that in healthy control subjects (Fig. 2A). Receiver operating characteristic (ROC) curves were generated for miR-223-3p expression, and the sensitivity and specificity were calculated based on the ROC curves. The sensitivity and specificity of serum miR-223-3p were determined by constructing an ROC curve with an Area Under the Curve (AUC) of 0.845, revealing a relatively high diagnostic power in distinguishing AR patients from healthy controls (Fig. 2B). These results collectively imply that miR-223-3p might be a potential positive regulator of the pro-allergic factors of AR and a promising clinical biomarker in patients with AR.

### Differential expression of miR-223-3p in the nasal mucosa of AR mice

**Figure 3:**
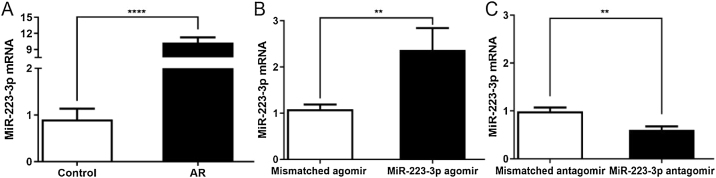
Expression of miR-223-3p in nasal mucosa of AR mice following intranasal administration of mismatched agomir/miR-223-3p agomir/mismatched antagomir/miR-223-3p antagomir. AR mice were induced as described in a previously published protocol and then intranasally treated with mismatched agomir/miR-223-3p agomir/mismatched antagomir/miR-223-3p antagomir after the last OVA challenge for one week. The expression of miR-223-3p in nasal mucosa was determined by qRT-PCR. (**p < 0.01, ****p < 0.0001).

To investigate the effects of miR-223-3p on the allergic response in AR, we overexpressed or downregulated miR-223-3p in the nasal mucosa of AR mice by intranasal administration of miR-223-3p agomir or miR-223-3p antagomir. As shown in Fig. 3, miR-223-3p expression was significantly elevated in AR mice compared with the control group and even increased slightly in miR-223-3p agomir-treated AR mice. In contrast, miR-223-3p expression was much lower in mice after nasal administration of miR-223-3p antagomir than in AR mice. These results suggest that miR-223-3p might facilitate AR and provide evidence for successful gene transduction in these mice.

### Expression of OVA-specific IgE and allergic symptoms in AR mice

**Figure 4:**
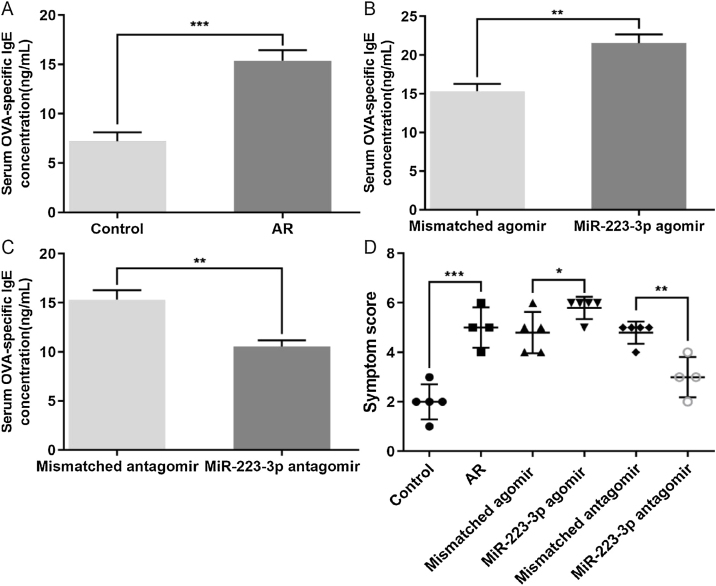
Effects of miR-223-3p on OVA-specific IgE and allergic symptoms in AR mice. (A) OD values of OVA-specific IgE in the serum of both the control and AR groups were measured using ELISA. (B) OD values of OVA-specific IgE in serum of the mismatched agomir and miR-223-3p agomir groups were measured using ELISA. (C) OD values of OVA-specific IgE in serum of the mismatched antagomir and miR-223-3p antagomir groups were measured using ELISA. (D) The symptom scores of the above three groups were calculated. The error bar indicates the SD. (*p < 0.05, **p < 0.01, ***p < 0.001).

We also evaluated the effects of miR-223-3p on the allergic response in AR mice by measuring OVA-specific IgE in the serum of these mice. The concentration of OVA-specific IgE was significantly increased in AR mice compared with normal mice (Fig. 4A). Furthermore, upregulation of miR-223-3p levels via intranasal administration of miR-223-3p agomir increased the OVA-specific IgE concentration, which was much lower in the miR-223-3p antagomir-treated group (Fig. 4 B,C). In addition, the nasal allergic symptoms, including the occurrences of nasal rubbing and sneezing, were counted within 30 min of the last OVA challenge on day 34. As displayed in Fig. 4D, the symptom score increased markedly in AR mice compared with normal mice and even became significantly higher in the miR-223-3p agomir-treated AR mice. The symptom score appeared to decrease in mice treated with miR-223-3p antagomir.

### MiR-223-3p upregulation increases the levels of cytokines in AR mice

**Figure 5:**
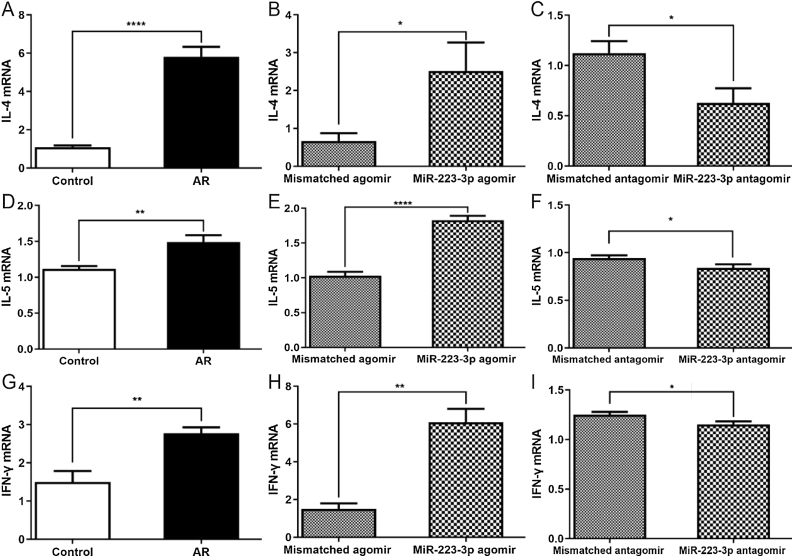
MiR-223-3p increases cytokine levels in AR mice. The mRNA expression levels of IL-4 (A‒C), IL-5 (D‒F), and IFN-γ (G‒I) were detected by qRT-PCR. Statistical significance was assessed using two-tailed Student’s *t*-test. The error bar indicates the SD. (*p < 0.05, **p < 0.01, ****p < 0.0001).

A previous study reported that intranasal administration of the OVA antigen could induce a Th2 lymphocyte response in the nasal mucosa, which generated IL-5 through the activation of innate lymphoid cells.^23^ Thus, we next investigated whether the upregulation of miR-223-3p increases Th2 immunity to OVA in vivo. Not surprisingly, the nasal mucosa tissues of AR mice produced higher Th2 cytokines (IL-4, IL-5) and higher Th1 cytokines (IFN-γ). Furthermore, all these cytokine levels increased in miR-223-3p agomir-treated AR mice compared with untreated AR mice (Fig. 5A,B, D,E, G,H). The mRNA expression levels of IL-4, IL-5 and IFN-γ were attenuated by miR-223-3p antagomir administration (Fig. 5 C, F and I).

### Promoting effect of miR-223-3p on the infiltration of inflammatory cells into nasal mucosa tissues

**Figure 6:**
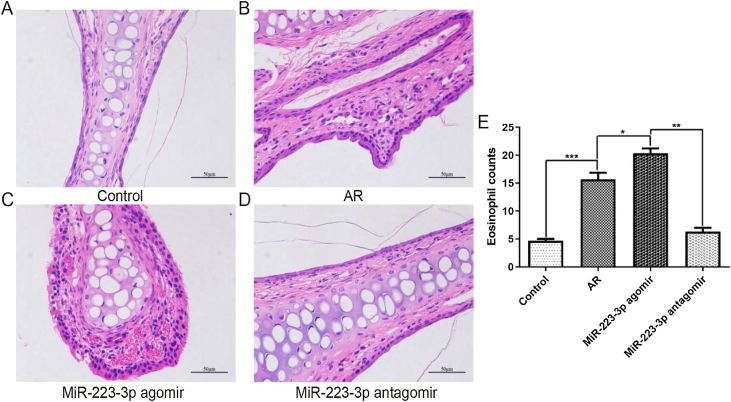
MiR-223-3p promoted an allergic inflammatory response in the nasal mucosa of AR mice. (A–D) The pathological changes in nasal mucosa from each group were analyzed using H&E staining. (E) The number of eosinophils in each group was quantified. (*p < 0.05, **p < 0.01, ***p < 0.001.

To further confirm the effect of miR-223-3p on the pathological alterations in the nasal mucosa of AR mice, paraffin-embedded tissue sections were examined using H&E staining. Consistent with the above results, OVA sensitization induced apparent pathological changes in nasal mucosal epithelium. Significantly worse hyperemia, edema, and chaotic structure existed in the nasal mucosal epithelium of AR mice than in normal control mice (Fig. 6 A,B). Furthermore, all these pathological alterations were aggravated after miR-223-3p agomir administration (Fig. 6C), and miR-223-3p antagomir ameliorated the allergic inflammation (Fig. 6D). To explore the effect of miR-223-3p on the infiltration of inflammatory cells into the nasal mucosa of AR, eosinophil cells were subsequently counted. We observed significantly more eosinophil cells in the nasal mucosa in AR mice than in control mice. Furthermore, intranasal administration of miR-223-3p agomir markedly increased the number of eosinophil cells, which was reduced in the miR-223-3p antagomir-treated group (Fig. 6E). These data demonstrated that miR-223-3p could promote allergic pathology with immune cell infiltration in the nasal mucosa of AR mice.

### Verification of INPP4A as a target gene of MiR-223-3p

**Figure 7:**
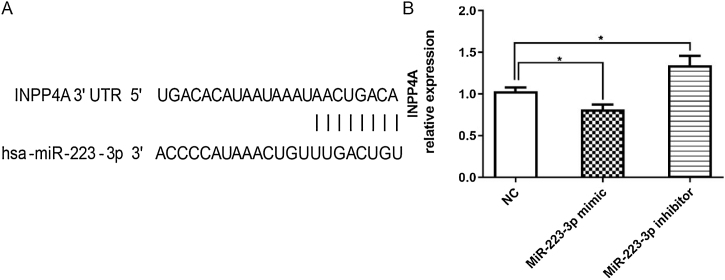
MiR-223-3p negatively regulates INPP4A expression. (A) The schematic of INPP4A mRNA shows a potential site in the INPP4A 3′-UTR for miR-223-3p. (B) qRT-PCR analysis of INPP4A mRNA levels in HEK293T cells transfected with miR-223-3p mimic or inhibitor. Statistical significance was assessed using Tukey’s multiple comparison post hoc test. The error bar indicates the SD. (*p < 0.05).

To further investigate the mechanisms by which miR-223-3p promoted allergic responses and symptoms in AR mice, potential target genes of miR-223-3p were predicted using the miRNA target gene prediction databases TargetScan (http://www.targetscan.org/vert_71/), miRWalk (http://mirwalk.umm.uni-heidelberg.de/) and miRDB (http://www.mirdb.org/). Notably, it was predicted that miR-223-3p might directly target inositol polyphosphate 4 phosphatase (INPP4A), a gene known to be involved in asthma and allergic disorders.^24^ As shown in Fig. 7A, an 8-bp fragment of the 3′UTR of the INPP4A gene is complementary to the miR-223-3p seed sequence. To verify this prediction, successful overexpression or knockdown of miR-223-3p in HEK293 T cells was verified by quantitative real-time PCR. As shown in Fig. 7B, overexpression of miR-223-3p by transfection of miR-223-3p mimics markedly decreased the mRNA expression of INPP4A, which was remarkably restored via transduction of miR-223-3p inhibitor. These results suggest that miR-223-3p may promote allergic inflammatory responses by negatively regulating INPP4A expression.

## Discussion

In the past few decades, miRNAs have emerged as critical regulatory molecules thought to be involved in the pathogenesis of various diseases.^25^, ^26^ They function by degrading their cognate targets and controlling gene expression at the posttranscriptional level by binding the 3′UTRs of their target mRNAs.^27^ Studies have shown that the stability of miRNAs could be exploited to clinically modulate gene expression in several diseases, including AR.^26^, ^28^ For example, miRNA-200a affected the proliferation of airway smooth muscle cells and airway remodeling by targeting FOXC.^29^ Moreover, overexpressed miRNA-485 modulated the TGF-β/Smads signaling pathway in chronic asthmatic mice by targeting Smurf2.^30^ In addition, miRNA-155 plays critical roles in the expression of Th2 factors and the allergic inflammatory response in allergic rhinitis.^31^ To our knowledge, the present study is the first to demonstrate that miR-223-3p suppresses INPP4A expression and promotes allergic inflammation in AR model mice.

A recent study demonstrated that miR-223 affects the PI3K/Akt signaling pathway by targeting IGF-1R and may become a promising target for the future treatment of asthma and airway remodeling.^32^ Moreover, miR-223-3p was reported to promote apoptosis and inhibit proliferation of hep3B cells by regulating NLRP3.^33^ Furthermore, miRNA-133b was also found to ameliorate allergic inflammation and symptoms in a murine model of allergic rhinitis by targeting Nlrp3.^21^ In addition, miR-223-3p expression was found to be higher in the synovium of Rheumatoid Arthritis (RA) patients than in the synovium of patients with osteoarthritis due to the increased number of miR-223-3p-positive cells.^34^ T-cells from RA patients also have higher miR-223-3p levels.^35^, ^36^ In accordance with a previous study, serum miR-223-3p levels were found to be increased in patients with AR compared with healthy individuals in our research. These results further suggested that miR-223-3p may positively regulate the pro-allergic properties of AR. Among the AR patients from whom samples were obtained, approximately 4.9% had a history of asthma, which accounted for a very low proportion. They were not experiencing an asthmatic attack at the time of sampling, and the interval between attacks and sampling was very long (more than two years for all patients). Therefore, asthma is not considered to have an external impact on the overall results of our study. Thus, we administered miR-223-3p agomir or antagomir into the nasal cavities of AR model mice and investigated the important effects of miR-223-3p on the symptoms and pathogenesis of AR. As expected, miR-223-3p expression was remarkably promoted after administration of miR-223-3p agomir. Furthermore, it was determined that the serum concentration of OVA-specific IgE and frequencies of rubbing and sneezing were higher in AR mice than in the control mice, and these manifestations were much more serious in miR-223-3p agomir-treated mice than in AR model mice, indicating that intranasal administration of miR-223-3p agomir may in fact promote the allergic response of AR. In contrast, the levels of OVA-specific IgE and the symptom scores were much lower in miR-223-3p antagomir-treated mice than in AR model mice, which indicates that intranasal administration of miR-223-3p antagomir may alleviate the pathology and symptoms in AR.

As AR is an inflammatory disorder of the nasal mucosa, researchers have found that many inflammatory cells, such as basophilic mast cells and eosinophils infiltrate the upper airways.^27^, ^37^ In addition, late-phase reactions in allergic inflammation are regulated by eosinophils. Infiltration by these inflammatory cells leads to the production of many proinflammatory cytokines, leukotrienes and histamine, thus triggering allergic symptoms in AR. Importantly, Th2-mediated immunological responses result in allergic inflammatory diseases, and the allergic inflammation typical of AR could be inhibited by the depletion of Th2 cells. A recent study from Schwindt et al.^38^ described that Th2 cytokines, such as IL4 and IL5, act as regulators of the differentiation of T cells and the switch of the IgE isotype in B cells. In addition, the authors found that Th1 cytokines (IFN-γ) may activate the production of Th2 cell cytokines in the allergic response. In this study, cytokines (IL-4, IL-5, IFN-γ) derived from Th1 or Th2 cells were increased in AR, thereby playing important roles in regulating immunological responses. Here, we found that the upregulated levels of cytokines (IL-4, IL-5, IFN-γ) by intranasal administration of miR-223-3p agomir were reversed by miR-223-3p antagomir. Furthermore, we found that intranasal administration of miR-223-3p agomir stimulates eosinophil infiltration in the nasal mucosa of AR mice, while miR-223-3p antagomir inhibits these pathological changes. These results suggest that miR-223-3p may be involved in regulating the proinflammatory effects of AR.

Previous studies have demonstrated that polymorphisms in the INPP4A gene are associated with asthma in humans, possibly due to reduced protein stability.^39^ In addition, INPP4A has also been shown to be a crucial molecular checkpoint in controlling the PI3K-Akt signaling pathway and is downregulated in allergic airway inflammation.^40^ Moreover, INPP4A is involved in the regulation of atopic asthma and may open new avenues for therapeutic intervention by modulating the PI3K-Akt pathway.^24^ Importantly, in this study, our data revealed that miR-223-3p affected INPP4A expression by directly binding to the 3′UTR of INPP4A. HEK293T cells transected with miR-223-3p mimic decreased the expression of INPP4A, while miR-223-3p inhibitor increased the above gene expression, suggesting that miR-223-3p promotes allergic inflammation by inhibiting INPP4A activation in AR mice. Although we have confirmed the targeting effect of miR-223-3p on INPP4A expression at the cell level, we will further determine the relationship between miR-223-3p and INPP4A by other in vivo assays. As miRNAs are directly responsible for several different aspects, such as inflammation, cell cycle, apoptosis, and cell migration, INPP4A is only one of the possible mechanisms. Thus, we will also study the key role of miRNAs in the mechanism of AR from other aspects.

## Conclusion

In conclusion, our results suggest that miR-223-3p plays a central role in the onset and development of allergic airway inflammation. MiR-223-3p overexpression markedly stimulates the pathology of AR through promotion of inflammatory responses, which include increased serum OVA-specific IgE concentration, eosinophil infiltration and proinflammatory cytokine levels, while downregulation of miR-223-3p had an anti-inflammatory effect via negative regulation of INPP4A. Overall, our findings reveal that miR-223-3p plays an important role in mediating allergic inﬂammation by targeting INPP4A.

## Funding

This study was supported by the Nantong Science and Technology Project Grant (nº MS12018071), the Jieping Wu Medical Foundation Clinical Research Project Grant (nº 320.6750.18272) and the Scientific Research Project of Jiangsu Health and Family Planning Commission Grant (nº Y2018100).

## Conflicts of interest

The authors declare no conflicts of interest.
